# 1,1,1,5,5,5-Hexafluoro-2,4-dimeth­oxy­pentane-2,4-diol

**DOI:** 10.1107/S1600536811044813

**Published:** 2011-11-02

**Authors:** Renier Koen, Andreas Roodt, Hendrik G. Visser, Theunis J. Muller

**Affiliations:** a Department of Chemistry, University of the Free State, PO Box 339, Bloemfontein 9300, South Africa

## Abstract

The title compound, C_7_H_10_F_6_O_4_, was isolated as an unexpected product from a reaction of tantalum(V) methoxide with hexa­fluoro­acetyl­acetone in a methanol solution. The asymmetric unit consists of one half-mol­ecule with the middle C atom lying on a twofold axis. The crystal structure is stabilized by O—H⋯O and an array of C—H⋯F hydrogen-bonding inter­actions. These inter­actions link the mol­ecules into a stable supra­molecular three-dimensional network. The mol­ecules pack in a ribbon-like form in the *ac* plane as a result of these inter­actions.

## Related literature

For metal complexes with acetyl­acetone derivatives, see: Viljoen *et al.* (2010 ▶); Steyn *et al.* (2008 ▶); Cole *et al.* (2005 ▶).
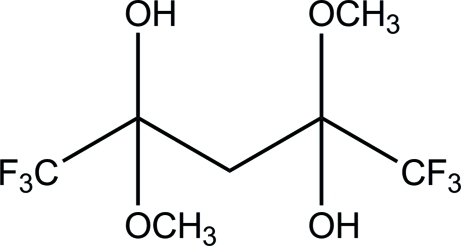


## Experimental

### 

#### Crystal data

C_7_H_10_F_6_O_4_
*M* *_r_* = 272.15
Monoclinic, 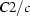

*a* = 17.829 (5) Å
*b* = 6.713 (5) Å
*c* = 9.347 (5) Å
β = 109.509 (5)°
*V* = 1054.5 (10) Å^3^
*Z* = 4
Mo *K*α radiation
μ = 0.20 mm^−1^
*T* = 100 K
0.75 × 0.28 × 0.19 mm

#### Data collection

Bruker APEXII CCD diffractometer
Absorption correction: multi-scan (*SADABS*; Bruker, 2007 ▶) *T*_min_ = 0.936, *T*_max_ = 0.963
5850 measured reflections
1277 independent reflections
1049 reflections with *I* > 2σ(*I*)
*R*_int_ = 0.025

#### Refinement

*R*[*F* ^2^ > 2σ(*F* ^2^)] = 0.030
*wR*(*F* ^2^) = 0.079
*S* = 1.05
1277 reflections
98 parameters
All H-atom parameters refined
Δρ_max_ = 0.39 e Å^−3^
Δρ_min_ = −0.24 e Å^−3^

### 

Data collection: *APEX2* (Bruker, 2007 ▶); cell refinement: *SAINT-Plus* (Bruker, 2007 ▶); data reduction: *SAINT-Plus*; program(s) used to solve structure: *SIR97* (Altomare *et al.*, 1999 ▶); program(s) used to refine structure: *SHELXL97* (Sheldrick, 2008 ▶); molecular graphics: *DIAMOND* (Brandenburg & Putz, 2005) ▶; software used to prepare material for publication: *WinGX* (Farrugia, 1999 ▶).

## Supplementary Material

Crystal structure: contains datablock(s) global, I. DOI: 10.1107/S1600536811044813/pv2464sup1.cif

Structure factors: contains datablock(s) I. DOI: 10.1107/S1600536811044813/pv2464Isup2.hkl

Supplementary material file. DOI: 10.1107/S1600536811044813/pv2464Isup3.cml

Additional supplementary materials:  crystallographic information; 3D view; checkCIF report

## Figures and Tables

**Table 1:** Hydrogen-bond geometry (Å, °)

*D*—H⋯*A*	*D*—H	H⋯*A*	*D*⋯*A*	*D*—H⋯*A*
O2—H2⋯O1^i^	0.835 (17)	1.935 (17)	2.6648 (14)	145.4 (16)
O2—H2⋯O2^ii^	0.835 (17)	2.640 (17)	3.073 (2)	113.7 (14)
C4—H4*A*⋯F3^iii^	0.954 (17)	2.741 (17)	3.644 (2)	158.4 (13)
C3—H3⋯F2^iv^	0.945 (13)	2.663 (13)	3.4022 (17)	135.5 (10)
C4—H4*A*⋯F1^v^	0.954 (17)	2.853 (17)	3.383 (3)	116.1 (11)

Symmetry codes: (i) 
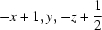
; (ii) 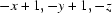; (iii) 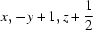; (iv) 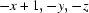; (v) 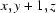.

**Fig. 1.**
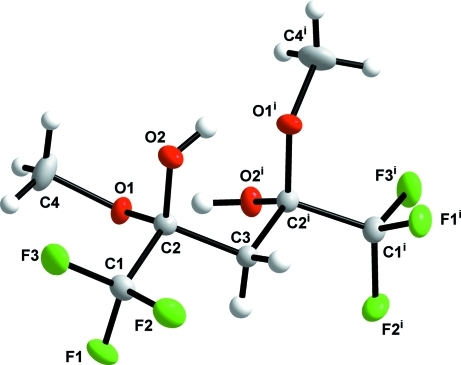
An ORTEP view of the title compound, showing the numbering scheme and displacement ellipsoids (50% probability). Symmetry code: i: 1 - x, y, 1/2 - z.

**Fig. 2.**
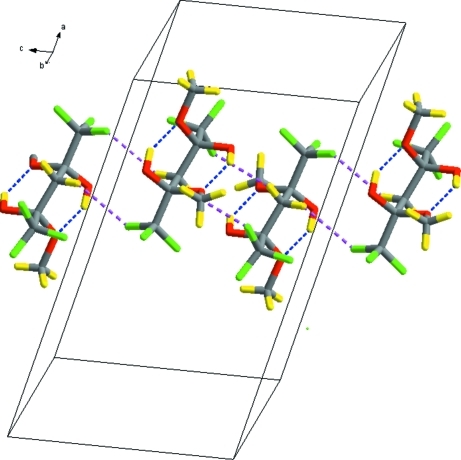
A partial packing diagram of the unit cell showing selected hydrogen bonding interactions of the title compound illustrating the ribbon-like formation across the ac plane.

## supplementary crystallographic information

### Comment

In continuation of our research on the formation kinetics of complexes derived from metals like hafnium, zirconium, etc., with different bidentate ligands (Viljoen *et al.*, 2010; Steyn *et al.*, 2008), an unexpected product, the title compound, was isolated after reacting tantalum(V) methoxide with hexafluoroacetylacetone in a methanol reaction solution.

The asymmetric unit of the title compound consists of a half molecule with C3 lying on a twofold axis (Figure 1). The bond angles and bond distances in the title compound are in accord with corresponding bond angles and distances reported for hexafluoroacetylacetone like derivatives (Cole *et al.*, 2005).

The crystal structure is stabilized by O—H···O (O···O separation 2.6648 (14) and 3.073 (2) Å) and an array of C—H···F (C···F separation in the range 3.383 (2)-3.644 (2)Å) hydrogen bonding interactions. All of these interactions serve to link the molecules into a stable supramolecular three-dimensional network. In the *ac* plane, the molecules pack in a ribbon-like formation as a result of these interactions (Figure 2).

### Experimental

The reaction was performed under modified Schlenk conditions under a nitrogen atmosphere. To a solution of Ta(OMe)_5_ (0.5010 g, 1.40 *x* 10 ^-3^ mol), a solution of hexafluoroacetylacetone (0.2912 g, 1.40 *x* 10 ^-3^ mol) was added and was placed in a sonic bath for 1 h. The resultant mixture was then stored at 252 K. After two days colourless crystals of the title compound were formed.

### Refinement

All H atoms were located from differencee Fourier maps and refined isotropically.

### Crystal data

**Table:**

C_7_H_10_F_6_O_4_	*F*(000) = 552
*M**_r_* = 272.15	*D*_x_ = 1.714 Mg m^−^^3^
Monoclinic, *C*2/*c*	Mo *K*α radiation, λ = 0.71073 Å
*a* = 17.829 (5) Å	Cell parameters from 2123 reflections
*b* = 6.713 (5) Å	θ = 3.3–28.1°
*c* = 9.347 (5) Å	µ = 0.20 mm^−^^1^
β = 109.509 (5)°	*T* = 100 K
*V* = 1054.5 (10) Å^3^	Needle, colourless
*Z* = 4	0.75 × 0.28 × 0.19 mm

### Data collection

**Table:**

Bruker APEXII CCD diffractometer	1049 reflections with *I* > 2σ(*I*)
graphite	*R*_int_ = 0.025
φ and ω scans	θ_max_ = 28°, θ_min_ = 3.8°
Absorption correction: multi-scan (*SADABS*; Bruker, 2007)	*h* = −23→21
*T*_min_ = 0.936, *T*_max_ = 0.963	*k* = −8→8
5850 measured reflections	*l* = −11→12
1277 independent reflections	

### Refinement

**Table:**

Refinement on *F*^2^	Primary atom site location: structure-invariant direct methods
Least-squares matrix: full	Secondary atom site location: difference Fourier map
*R*[*F*^2^ > 2σ(*F*^2^)] = 0.030	Hydrogen site location: inferred from neighbouring sites
*wR*(*F*^2^) = 0.079	All H-atom parameters refined
*S* = 1.05	*w* = 1/[σ^2^(*F*_o_^2^) + (0.0386*P*)^2^ + 0.4659*P*] where *P* = (*F*_o_^2^ + 2*F*_c_^2^)/3
1277 reflections	(Δ/σ)_max_ < 0.001
98 parameters	Δρ_max_ = 0.39 e Å^−^^3^
0 restraints	Δρ_min_ = −0.24 e Å^−^^3^

### Special details

**Table:**

Geometry. All s.u.'s (except the s.u. in the dihedral angle between two l.s. planes) are estimated using the full covariance matrix. The cell s.u.'s are taken into account individually in the estimation of s.u.'s in distances, angles and torsion angles; correlations between s.u.'s in cell parameters are only used when they are defined by crystal symmetry. An approximate (isotropic) treatment of cell s.u.'s is used for estimating s.u.'s involving l.s. planes.
Refinement. Refinement of *F*^2^ against ALL reflections. The weighted *R*-factor *wR* and goodness of fit *S* are based on *F*^2^, conventional *R*-factors *R* are based on *F*, with *F* set to zero for negative *F*^2^. The threshold expression of *F*^2^ > 2σ(*F*^2^) is used only for calculating *R*-factors(gt) *etc*. and is not relevant to the choice of reflections for refinement. *R*-factors based on *F*^2^ are statistically about twice as large as those based on *F*, and *R*- factors based on ALL data will be even larger.

### Fractional atomic coordinates and isotropic or equivalent isotropic displacement parameters (Å^2^)

**Table:**

	*x*	*y*	*z*	*U*_iso_*/*U*_eq_	
F1	0.67222 (4)	0.00201 (11)	0.36009 (8)	0.0251 (2)	
F2	0.61085 (5)	−0.06716 (11)	0.12475 (8)	0.0263 (2)	
F3	0.69105 (4)	0.18291 (12)	0.18451 (9)	0.0286 (2)	
O1	0.59145 (5)	0.34897 (12)	0.35035 (9)	0.0176 (2)	
O2	0.54269 (5)	0.31437 (13)	0.08511 (9)	0.0192 (2)	
C1	0.63589 (7)	0.08280 (19)	0.22390 (13)	0.0192 (3)	
C2	0.56606 (7)	0.21774 (18)	0.22550 (12)	0.0153 (3)	
C3	0.5	0.0899 (2)	0.25	0.0150 (3)	
C4	0.64796 (10)	0.5018 (2)	0.34926 (19)	0.0311 (3)	
H3	0.4785 (8)	0.008 (2)	0.1636 (14)	0.016 (3)*	
H4A	0.6446 (9)	0.598 (3)	0.4222 (18)	0.032 (4)*	
H4B	0.6369 (10)	0.559 (3)	0.251 (2)	0.043 (5)*	
H4C	0.7027 (13)	0.450 (3)	0.383 (2)	0.056 (6)*	
H2	0.4960 (10)	0.351 (3)	0.0706 (18)	0.036 (5)*	

### Atomic displacement parameters (Å^2^)

**Table:**

	*U*^11^	*U*^22^	*U*^33^	*U*^12^	*U*^13^	*U*^23^
F1	0.0227 (4)	0.0299 (4)	0.0202 (4)	0.0092 (3)	0.0038 (3)	0.0038 (3)
F2	0.0285 (4)	0.0259 (4)	0.0241 (4)	0.0068 (3)	0.0083 (3)	−0.0070 (3)
F3	0.0198 (4)	0.0341 (5)	0.0382 (5)	0.0039 (3)	0.0179 (3)	0.0044 (3)
O1	0.0171 (4)	0.0168 (4)	0.0207 (4)	−0.0048 (3)	0.0085 (3)	−0.0045 (3)
O2	0.0164 (5)	0.0238 (5)	0.0187 (4)	0.0031 (4)	0.0076 (3)	0.0064 (3)
C1	0.0181 (6)	0.0226 (6)	0.0171 (6)	0.0024 (5)	0.0062 (5)	−0.0007 (4)
C2	0.0159 (6)	0.0162 (5)	0.0143 (6)	0.0011 (4)	0.0057 (4)	0.0001 (4)
C3	0.0149 (8)	0.0148 (8)	0.0152 (8)	0	0.0049 (6)	0
C4	0.0336 (8)	0.0291 (8)	0.0342 (8)	−0.0165 (6)	0.0159 (7)	−0.0074 (6)

### Geometric parameters (Å, °)

**Table:**

F1—C1	1.3354 (15)	C1—C2	1.5438 (17)
F2—C1	1.3399 (16)	C2—C3	1.5352 (15)
F3—C1	1.3402 (15)	C3—C2^i^	1.5352 (15)
O1—C2	1.4107 (15)	C3—H3	0.945 (13)
O1—C4	1.4405 (17)	C4—H4A	0.954 (17)
O2—C2	1.3968 (15)	C4—H4B	0.950 (17)
O2—H2	0.835 (17)	C4—H4C	0.98 (2)
			
C2—O1—C4	118.10 (10)	O1—C2—C1	109.72 (9)
C2—O2—H2	104.9 (11)	C3—C2—C1	109.55 (11)
F1—C1—F2	107.32 (11)	C2—C3—C2^i^	112.00 (14)
F1—C1—F3	107.38 (10)	C2—C3—H3	108.1 (8)
F2—C1—F3	107.00 (10)	C2^i^—C3—H3	109.8 (8)
F1—C1—C2	111.41 (10)	O1—C4—H4A	105.3 (10)
F2—C1—C2	111.35 (10)	O1—C4—H4B	111.8 (11)
F3—C1—C2	112.12 (11)	H4A—C4—H4B	112.1 (14)
O2—C2—O1	113.56 (11)	O1—C4—H4C	111.7 (12)
O2—C2—C3	113.38 (9)	H4A—C4—H4C	107.4 (15)
O1—C2—C3	106.02 (9)	H4B—C4—H4C	108.4 (15)
O2—C2—C1	104.60 (9)		
			
C4—O1—C2—O2	−48.50 (14)	F3—C1—C2—O1	−71.61 (13)
C4—O1—C2—C3	−173.64 (11)	F1—C1—C2—C3	−67.23 (11)
C4—O1—C2—C1	68.14 (14)	F2—C1—C2—C3	52.54 (12)
F1—C1—C2—O2	170.92 (9)	F3—C1—C2—C3	172.38 (8)
F2—C1—C2—O2	−69.30 (12)	O2—C2—C3—C2^i^	−71.42 (8)
F3—C1—C2—O2	50.54 (12)	O1—C2—C3—C2^i^	53.84 (6)
F1—C1—C2—O1	48.77 (13)	C1—C2—C3—C2^i^	172.17 (10)
F2—C1—C2—O1	168.55 (9)		

Symmetry codes: (i) −*x*+1, *y*, −*z*+1/2.

### Hydrogen-bond geometry (Å, °)

**Table:**

*D*—H···*A*	*D*—H	H···*A*	*D*···*A*	*D*—H···*A*
O2—H2···O1^i^	0.835 (17)	1.935 (17)	2.6648 (14)	145.4 (16)
O2—H2···O2^ii^	0.835 (17)	2.640 (17)	3.073 (2)	113.7 (14)
C4—H4A···F3^iii^	0.954 (17)	2.741 (17)	3.644 (2)	158.4 (13)
C3—H3···F2^iv^	0.945 (13)	2.663 (13)	3.4022 (17)	135.5 (10)
C4—H4A···F1^v^	0.954 (17)	2.853 (17)	3.383 (3)	116.1 (11)

Symmetry codes: (i) −*x*+1, *y*, −*z*+1/2; (ii) −*x*+1, −*y*+1, −*z*; (iii) *x*, −*y*+1, *z*+1/2; (iv) −*x*+1, −*y*, −*z*; (v) *x*, *y*+1, *z*.
